# Comparison of APACHE II and Imrie Scoring Systems in predicting the severity of Acute Pancreatitis

**DOI:** 10.1186/1749-7922-2-33

**Published:** 2007-12-09

**Authors:** Savio G Barreto, Jude Rodrigues

**Affiliations:** 1 Department of General Surgery, Goa Medical College, Bamboliim, India

## Abstract

There has been an increasing amount of work worldwide in search for tests not only to be able to absolutely diagnose acute pancreatitis, but more importantly to prognosticate patients at admission. While the tests are still within the realm of research laboratories and involve complex computing and analytical methods, we believed that the already widely practiced methods of scoring needed to be verified in the Indian context. And, hence, the study.

## Letter to Editor

Being able to predict the prognosis of a patient with acute pancreatitis at admission forms a very important strategy considering that this will enable us to practice guidelines for standardization of management of the patient, viz., the use of antibiotics, timings of computed tomography scans, use of ERCP and operative intervention. This will in turn translate into improved outcomes [1]. Data on the use of scoring systems in India and Asia, as a whole, are sparse. The aim of this study was to compare the accuracy of APACHE II and Imrie scoring systems in assessing severity of acute pancreatitis.

All patients who presented to a tertiary care referral centre with a diagnosis of acute pancreatitis between June 2003 and January 2005 were prospectively evaluated.

The diagnostic criteria used for acute pancreatitis included:

1) Clinical criteria – history of pain in abdomen radiating to the back and relieved on bending forward associated with tenderness/guarding in the upper abdomen.

2) Radiographic evidence – Computed Tomography findings suggestive of acute pancreatitis such as pancreatic edema, pancreatic necrosis, peripancreatic fluid collections

3) Biochemical – Serum amylase concentration greater than 180 Somogyii units (by the Somogyii method).

The Atlanta Consensus definitions of severe and mild disease were used [2]. Acute pancreatitis was classified as severe if the patient had associated organ failure and/or local complications such as necrosis, abscess, or pseudocyst. The episode was also labeled severe if the patient required surgical intervention. If the episode was associated with minimal organ dysfunction and uneventful recovery without the features considered under severe acute pancreatitis, it was deemed to be mild.

Presentation data on admission and at 48 hours were collected. Acute Physiology and Chronic Health Evaluation (APACHE) II and Imrie scores were calculated within the first 48 hours of admission.

The APACHE II scoring system as proposed by Knaus et al. [3] was used. Scores above 9 were considered indicative of severe disease.

*Modified Glasgow (Imrie's) Severity Criteria *(Severity is indicated if >3 criteria are detected within 48 hours of onset of attack)

• Age > 55 yrs

• WBC count > 15 × 10^9^/l

• Blood Glucose > 10 mmol/l

• Blood Urea > 16 mmol/l

• Arterial oxygen partial pressure <8.0 kPa

• Serum Albumin < 32 g/l

• Serum calcium < 2.0 mmol/l

• Lactate Dehydrogenase > 600 U/l

The sensitivities, specificities and accuracies of the scoring systems were compared.

A total of 282 patients were diagnosed with acute pancreatitis. These included 271 males (96.1%) and 11 females (3.9%). The median age was 40 years (range 23–80). Alcohol was found to be the predominant cause of acute pancreatitis accounting for 92.6% (260/282). Other aetiologies included biliary (19 patients), trauma (1 patient), idiopathic (1 patient), and ascariasis of the pancreatic duct (1 patient). Of the 282 patients, 189 patients (67%) had mild disease while 93 (33%) patients had severe disease. The overall mortality rate was 12% (34/282).

By the Imrie scoring system, 55 attacks were predicted severe on the initial Imrie prognostic score of which 52 patients actually had a severe outcome. Of the 227 attacks predicted as mild, 41 patients had a severe outcome. Thus the sensitivity, specificity, positive and negative predictive value were 56%, 98%, 94%, and 80%, respectively. The overall accuracy of the test was 84%.

By the APACHE II scoring system, 59 attacks were predicted severe based on the highest APACHE II score (at 48 hours) of which 56 patients actually had a severe outcome. Of the 223 attacks predicted as mild, 37 patients had a severe outcome. Thus the sensitivity, specificity, positive and negative predictive value were 56%, 98%, 95%, and 82%, respectively. The overall accuracy of the test was 84%.

To determine whether there was a significant difference between the two tests, the results for each test for all patients were tabulated in a 2 × 2 table and the Mac Nemar chi square test was applied. Applying Mac Nemar Chi square test, p = 0.557 (Not significant)

We compared our results with other similar studies from across the world [4-8]. Interesting to note are that our findings were quite consistent with those groups reporting alcohol as the predominant aetiology.

In the Imrie scoring system, when a score of ≥ 3 was used as a cut-off, the sensitivity seemed to be on the lower side (52–56%) [4]. This was true even in our study. However, the specificity, positive and negative predictive values are very acceptable. If, on the other hand, the cut-off value is kept at ≥ 2 [5-8], then the sensitivity improves. This occurs at the expense of the other measures.

In the case of the APACHE II scoring system, despite keeping our cut-off value at ≥ 9, it is interesting to note that the measures under study, viz. the sensitivity, specificity, positive and negative predictive values were comparable to Tran et al. [4]. These results were in stark contrast to those reported by Wilson et al. [5] (sensitivity = 82%, specificity = 74%, positive predictive value = 50% and negative predictive value = 93%) and Fan et al [6]. (sensitivity = 64%, specificity = 59%, positive predictive value = 33% and negative predictive value = 84%) who also used 9 as a cut-off.

Whether this reflects an aetiological influence on the results can be well regarded as a point of conjecture.

On comparing the two scoring systems, the APACHE II subjectively (p value – not significant) seems to be a better scoring system than Imrie. This benefit appears to arise from the fact that it is reproducible and convenient.

As newer systems of prognosticating patients are introduced, viz. artificial neural networks, they are yet under study and involve complex analytical methodology. This exercise has objectively demonstrated that the tests we have been using for scoring patients in most surgical centres in our country are indeed very useful and effective, and can continue to be used till such time as a simplistic and much more accurate system of scoring is devised.

## Competing interests

The author(s) declare that they have no competing interests.
